# Successful removal of a fractured guidewire from the main pancreatic duct using a novel endoscopic device

**DOI:** 10.1055/a-2713-3460

**Published:** 2025-10-29

**Authors:** Amer Al-Muzaini, Akihisa Ohno, Nao Fujimori, Yosuke Minoda, Keijiro Ueda, Tomohiko Moriyama, Yoshihiro Ogawa

**Affiliations:** 1 12923 Department of Medicine and Bioregulatory Science, Graduate School of Medical Sciences, Kyushu University, Fukuoka, Japan; 2 37853 Department of Gastroenterology, Prince Sultan Military Medical City, Riyadh, Saudi Arabia; 3 145181 International Medical Department, Kyushu University Hospital, Fukuoka, Japan

A guidewire fracture in the main pancreatic duct (MPD) is uncommon during endoscopic retrograde cholangiopancreatography (ERCP). The number of reported cases of fractured guidewires in the MPD is limited, and most retrieval attempts present significant technical challenges 1 2 . We used a novel endoscopic sheath (EndoSheather; Piolax Medical Devices, Kanagawa, Japan) with a tapered inner sheath to facilitate passage through strictures as a rescue technique. Instruments with lengths of up to 1.9 mm could be easily inserted after removal of the inner sheath. Several reports have described the use of this device to retrieve migrated stents 3 4 5 , but no previous publication has documented the retrieval of a fractured guidewire in the MPD ( Fig. 1 ).

**Fig. 1:**
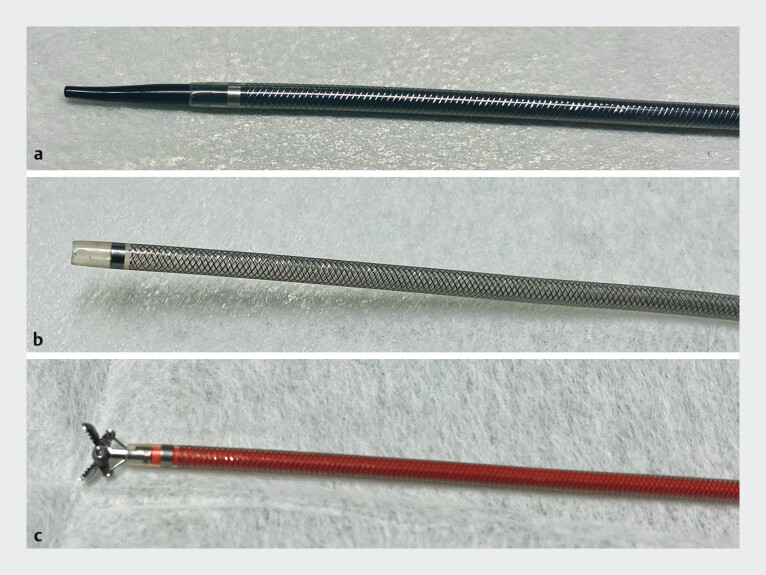
The novel endoscopic sheath system. **a** The sheath with both inner and outer components assembled. **b** The outer sheath remaining after the removal of the inner sheath. **c** Biopsy forceps inserted through the outer sheath following the removal of the inner sheath.

A 46-year-old man with a history of jaundice and abdominal pain initially underwent ERCP at a different institution for the removal of common bile duct stones. The catheter and guidewire were inadvertently advanced into the MPD during stone extraction, resulting in a guidewire tip fracture inside the MPD ( Fig. 2 **a** ). The fragment remained lodged in the MPD after multiple retrieval attempts, leading to acute pancreatitis ( Fig. 2 **b** ). A rescue ERCP was subsequently performed at our institution. Pancreatography revealed a focal stricture extending from the pancreatic head to the body, with mild upstream dilation in the tail ( Fig. 3 ). A novel device (EndoSheater) was inserted proximal to the retained guidewire. Biopsy forceps (Radial Jaw 4 Standard Capacity, Boston Scientific, Marlborough, MA, USA) were inserted through the outer sheath after the inner sheath was removed to grasp the guidewire. The fractured guidewire was successfully removed through the device without any complications ( Fig. 4 and Video 1 ). This case demonstrates the safe and effective retrieval of a fractured guidewire from the MPD using the novel endoscopic sheath.

**Fig. 2:**
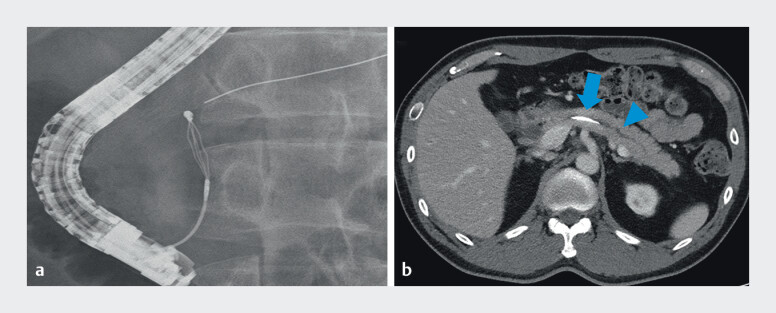
Fractured guidewire retained in the main pancreatic duct. **a** Fluoroscopic view revealed the fractured guidewire in the main pancreatic duct (MPD). **b** Contrast-enhanced computed tomography scan showing impacted guidewire (blue arrow) in the MPD with mild upstream dilation in the pancreatic tail (blue arrowhead).

**Fig. 3:**
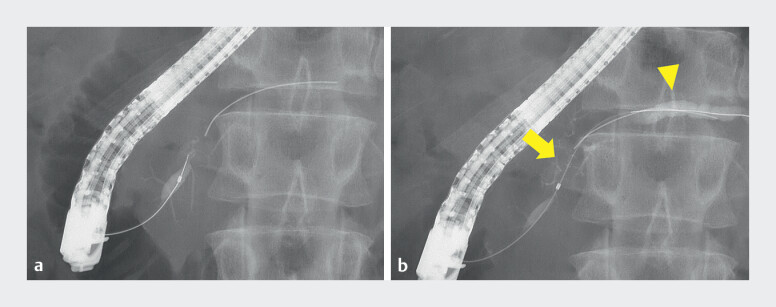
Fluoroscopic findings of main pancreatic duct (MPD) stricture and guidewire fracture. **a** Fluoroscopic view showing the initial assessment of the MPD and the location of the fractured guidewire. **b** Fluoroscopic view showing a focal stricture from the head to body of the MPD (yellow arrow) and upstream dilation in the pancreatic tail (yellow arrowhead).

**Fig. 4:**
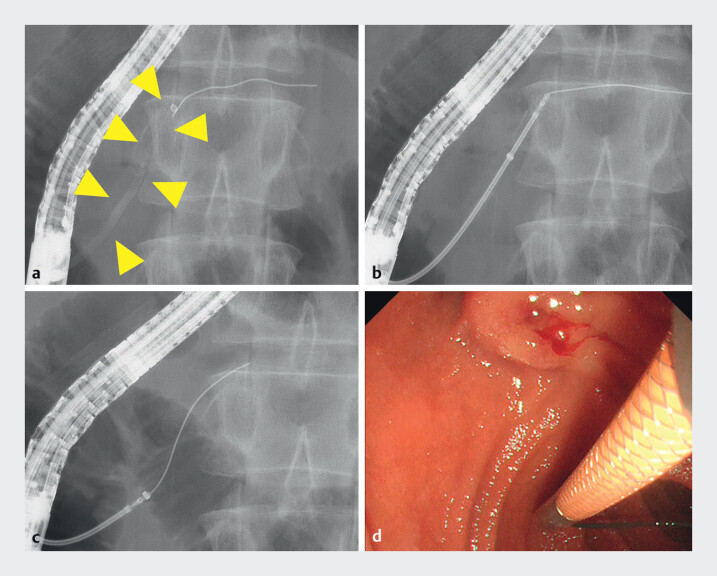
Retrieval of the fractured guidewire using EndoSheather. **a** Fluoroscopic view showing the EndoSheather having been passed through the stricture between head and body; image obtained after removal of the inner sheath (yellow arrowhead). **b** Fluoroscopic view showing the insertion of biopsy forceps through the EndoSheather to grasp the proximal end of the fractured guidewire. Fluoroscopic **c** and endoscopic **d** views showing successful and safe retrieval of the fractured guidewire.

Successful retrieval of a fractured pancreatic duct guidewire using a novel endoscopic sheath system.Video 1

Endoscopy_UCTN_Code_CPL_1AK_2AD

## Notes

Endoscopy E-Videos https://eref.thieme.de/e-videos
*E-Videos* is an open access online section of the journal *Endoscopy* , reporting on interesting cases and new techniques in gastroenterological endoscopy. All papers include a high-quality video and are published with a Creative Commons CC-BY license. Endoscopy E-Videos qualify for HINARI discounts and waivers and eligibility is automatically checked during the submission process. We grant 100% waivers to articles whose corresponding authors are based in Group A countries and 50% waivers to those who are based in Group B countries as classified by Research4Life (see: https://www.research4life.org/access/eligibility/ ). This section has its own submission website at https://mc.manuscriptcentral.com/e-videos .
